# Factors influencing patients’ experience of communication with the medical team of the emergency department

**DOI:** 10.1007/s11739-023-03298-5

**Published:** 2023-05-04

**Authors:** Dea Degabriel, Roberta Petrino, Eleonora Dafne Frau, Laura Uccella

**Affiliations:** 1 Internal Medicine Department, EOC-Ospedale Regionale di Lugano, via Tesserete 46, 6900 Lugano, Switzerland; 2 Emergency Department, EOC-Ospedale Regionale di Lugano, via Tesserete 46, 6900 Lugano, Switzerland

**Keywords:** Communication, Emergency department, Doctor–patient relationship, Perception, CAT-T

## Abstract

The Emergency Department (ED) setting often presents situations where the doctor–patient relationship is fundamental and may be challenging. Thus, it is important to use effective communication to improve outcomes. This study explores patients’ experience of communication with the medical team aiming to discover whether there are some objective factors which may affect their perception. A prospective, cross-sectional study in two hospitals: an urban, academic trauma center and a small city hospital. Adult patients discharged from the ED in October 2021 were consecutively included. Patients filled out a validated questionnaire, the Communication Assessment Tool for Teams (CAT-T), assessing communication perception. Additional data about the participants were collected by the physician in a dedicated tab to assess whether there were objective factors influencing the patient’s perception of the medical team's communication skills. Statistical analysis was then performed. 394 questionnaires were analyzed. The average score for all items exceeded 4 (good). Younger patients and patients who were conveyed by ambulance attributed lower scores than other groups (*p* value < 0.05). A significant difference between the two hospitals was also observed in favour of the bigger hospital. In our study long waiting times did not generate less satisfied responses. The item which received the lowest scores was “the medical team encouraged me to ask questions”. Overall, patients were satisfied with doctor–patient communication. Age, setting, way of conveyance to the hospital are objective factors that may influence patients’ experience and satisfaction in the ED.

**Supplementary Information:**

The online version contains supplementary material available at 10.1007/s11739-023-03298-5.

## Introduction

“The patient will never care how much you know, until they know how much you care” [1, 2]. What is a doctor’s most important task? To recognize signs and symptoms of a disease, postulate a diagnosis and prescribe the appropriate treatment. This, however, is not enough: it is also necessary to empathise with the patient, listen, take an interest to her/his story and needs to establish the therapeutic alliance [3, 4].

The doctor–patient relationship is a big challenge a doctor has to face. This relationship can be as intense as it is fragile but indispensable: only once it is established, can the doctor and the patient begin the diagnostic and therapeutic process towards full ‘caring’. How can such an important and delicate relationship be built during an Emergency Department (ED) visit? In the ED, time is precious and runs fast.

Communication in the ED is important for providing quality care.

Communication skills (verbal and non-verbal), empathy, and a patient-centred approach by the medical and nursing team have a great impact on patient experience and satisfaction [5–7]. Improved skills can lead to an increased therapeutic efficacy, improved outcomes and patient safety, as well as increased adherence to prescribed therapy [7, 8].

The literature has already established the correlation between effective doctor–patient communication and the achievement of better outcomes for the patient in terms of patient satisfaction, improved compliance and therapeutic adherence, more robust exchange of information, reduction of conflicts and medical errors, improvement of the working environment and decreased stress for the emergency team, reduction of waiting times, avoidance of repeated visits and examinations for the same reason, with consequent reduction of the associated costs and associated risks [8–12].

The ED is a particular and difficult setting as it offers many communication challenges: the need for multiple interactions between different professionals, frequent interruptions, limited time, lack of a pre-existing doctor–patient relationship, long waiting times [13].

In the literature there are many qualitative studies on communication in the ED. However, very few works offer quantitative data on this topic.

Data on whether there are extrinsic, objective factors influencing patients' perception of communication are missing.

This study aims to answer the question whether the patients who come to the ED are satisfied with the doctor–patient communication and whether there are some objective factors which may affect the quality of communication perceived by patients.

## Patients and methods

### Study design

This is an observational, prospective, cross-sectional study conducted during a one month period in October 2021 in an urban, academic, level 1 trauma center (Hospital A) with around 30,000 ED visits/year in the south of Switzerland. In parallel, the study was administered in a second, smaller site (Hospital B), with around 8000 visits/year pertaining to the same administration.

Hospital A is a facility with intensive care, surgery, invasive cardiology, interventional angiography, neurosurgery, a stroke unit and all the various specialities, whereas hospital B is a facility dedicated mainly to the care of outpatients.

Between the 2 hospitals there are also other differences, in particular in terms of the buildings (Hospital B is of more recent construction) and geographical location (Hospital B in the city center, while Hospital A is more peripheral).

The medical, nursing and administrative teams, however, are the same. The workers are, therefore, regularly employed in both sites, based on their monthly shift plans.

All adults (> 18 years-old) who were discharged by the ED were eligible for the study, if the following exclusion criteria did not apply: critically ill, with psychiatric disease or cognitive disorder (e.g., dementia) and non-Italian speaking. Patients were enrolled consecutively, without any selection.

Upon ED discharge, patients were asked to fill out the Italian version of the CAT-T, a validated instrument for measuring patients’ perceptions of physicians’ performance in the area of interpersonal and communication skills [14–17].

The CAT-T is a 15-item survey measuring responses on a 5-point scale (1 = poor, 5 = excellent). Given that the 14 core items of the survey focus on communication within the doctor–patient relationship, the additional item number 15 (“the staff treated me with respect”) was excluded from this analysis because it was not related to the focus of the study.

The CAT -T was psychometrically validated to measure patient perceptions of communication with medical teams and has already been used in the emergency care setting [14–17]. Scale development processes and psychometric properties are detailed in the original CAT article [14].

Additional data about the participants were collected by the physician in a dedicated tab: gender, age, medical category (surgical or medical), time of arrival at the ED, time of entry into the ED bay, time of discharge from the ED, total ED length of stay, holiday or working day, type of convey to the ED (family-doctor, ambulance, patient’s own initiative); the hospital site (A/B); function held by the doctor/s who examined the patient (resident, senior or both) (see Table 1).

**Table 1:** Characteristics of patients who completed the CAT-T (*n* = 394)

Characteristics	*N*	%
Age		
< 30 y/o	89	22.6
> 30–64 y/o	218	55.3
> 65–79 y/o	65	16.5
> 80 y/o	22	5.6
Medical category		
Surgery	230	58.4
Medicine	164	41.6
Day		
Working day	284	72.1
Holiday/week-end	110	27.9
Time spent in the ED		
< 1 h	110	27.9
1–3 h	190	48.2
3–6 h	84	21.3
> 6 h	10	2.5
Time spent waiting		
< 30 min	271	68.8
30-60 min	60	15.2
> 60 min	63	16
Time spent in ED bay		
< 30 min	100	25.4
30-60 min	72	18.3
> 60 min	222	56.4
Means of conveyance to the ED		
Announced by family physician	19	4.8
Ambulance	25	6.4
By own initiative	350	88.8
Hospital site		
Hospital A	251	63.7
Hospital B	143	36.3
Physician function		
Assistant in training	203	51.5
Senior	97	24.6
Assistant in training AND senior	94	23.9

These data were collected for the purpose of correlating, where possible, patients’ perceptions of medical communication with objective factors dependent on the patients’ biographical characteristics, the type of doctor visiting them, waiting times, total length of stay, and how they arrived at the hospital.

Patients were asked to participate in the study while they were about to leave the ED, after a brief oral presentation of the study by the doctor who had treated them. The questionnaire was anonymous. The additional data collected were not connected with patient or physician names and all patients provided verbal consent before study enrolment. The regional Ethics Committee (Cantonal Ethics Committee of Ticino) approved this research project without requiring formal written consent because consent was implied by completion of the questionnaire.

### Statistical analysis

Statistical analysis was performed using the open source packages “Pandas”, “NumPy”, “SciPy”, “Seaborn” and “PyMC” for Mac Os X versions 1.4.1, 1.21.2, 1.7.3, 0.11.2 and 3.11.14, respectively. Statistical significance was considered achieved based on highly credible intervals of parameter estimates. Confidence intervals (CI) were calculated at 95%.

As the number of unsatisfied responders was small, we used a Bayesian methodology (albeit with uninformative a-priori) because it does not have the sample size limitations that frequentist methods relying on asymptotics have. Bayesian methodology addresses uncertainty by producing “wider” Confidence Intervals. This means that when the data are many the CI is narrow, while if the data are few the range widens. If, therefore, the CI of two different subgroups are well separated (e.g., young vs. old), the data assumes strong statistical significance.

We calculated a *P* value only as a usual reference.

The problem with measuring the difference in scores was that the scores (assigning a numerical score to a qualitative judgment such as “excellent” or “poor”) are ordinal but not metric measures. Averaging the values obtained in this way is not meaningful because variables that are neither quantitative nor metric are measured. We decided, for each item of the questionnaire, to divide the scores into “satisfied” and “non-satisfied” patients. Only scores of “5” (excellent) were considered a satisfied answer, and all the answers below 5 were considered non-satisfied. We then measured the proportion of people who gave a satisfied answer in the questionnaire and compared it with the proportion of people who gave a non-satisfied answer.

We considered that those who were totally satisfied with their experience of communication with doctors in the ED, would rate the items with the highest score (5), and those who perceived even a small aspect which failed to satisfy them completely, would give a score below 5.

## Results

During the study period (October 1–31, 2021), a total of 1753 patients were discharged from our ED (1357 from Hospital A and 396 from Hospital B).

A total of 432 patients were enrolled for the study. Nine patients were excluded from the initial analysis because they lacked demographic and operational data (age, gender, medical category, wait time, etc…). Another 29 patients were subsequently excluded because they did not return the questionnaire, so statistics were made on a sample of 394 patients.

That means that about 1/4 of the discharged patients completed the questionnaire. The characteristics of patients who responded to the questionnaire did not differ significantly in age, gender, type of presented complaint, from the population of all patients discharged from ED during the examined period.

Characteristics of the final sample are shown in Table 1. Complete datasheet is available in Online Resource 1.

Scores obtained indicated that overall, patients were satisfied with doctor–patient communication. Mean scores, standard deviations and proportion of completely satisfied patients are shown in Table 2.

**Table 2:** Mean scores, standard deviations and % of satisfied scores (= 5) per question

Questions	Mean	Standard deviation	Score = 5 (%)
1. Greeted me in a way that made me feel comfortable	4.745257	0.5418723	73.6%
2. Treated me with respect	4.81586	0.5023836	80.4%
3. Showed interest in my ideas about my health	4.747967	0.5664079	74.8%
4. Understood my main health concerns	4.710027	0.5954376	71.8%
5. Paid attention to me (looked at me, listened carefully)	4.773842	0.5087456	75.3%
6. Let me talk without interruptions	4.817568	0.4797691	79.4%
7. Gave me as much information as I wanted	4.746612	0.5381083	74.1
8. Talked in terms I could understand	4.794595	0.4782227	77.4%
9. Checked to be sure I understood everything	4.722071	0.5572454	71.5%
10. Encouraged me to ask questions	4.461538	0.8248241	57.8%
11. Involved me in decisions as much as I wanted	4.577135	0.7232145	63.4%
12. Discussed next steps, including any follow-up plans	4.646893	0.6580544	65.2%
13. Showed care and concern	4.684282	0.6351755	70.8%
14. Spent the right amount of time with me	4.664835	0.5768943	70.0%

**Fig. 1:**
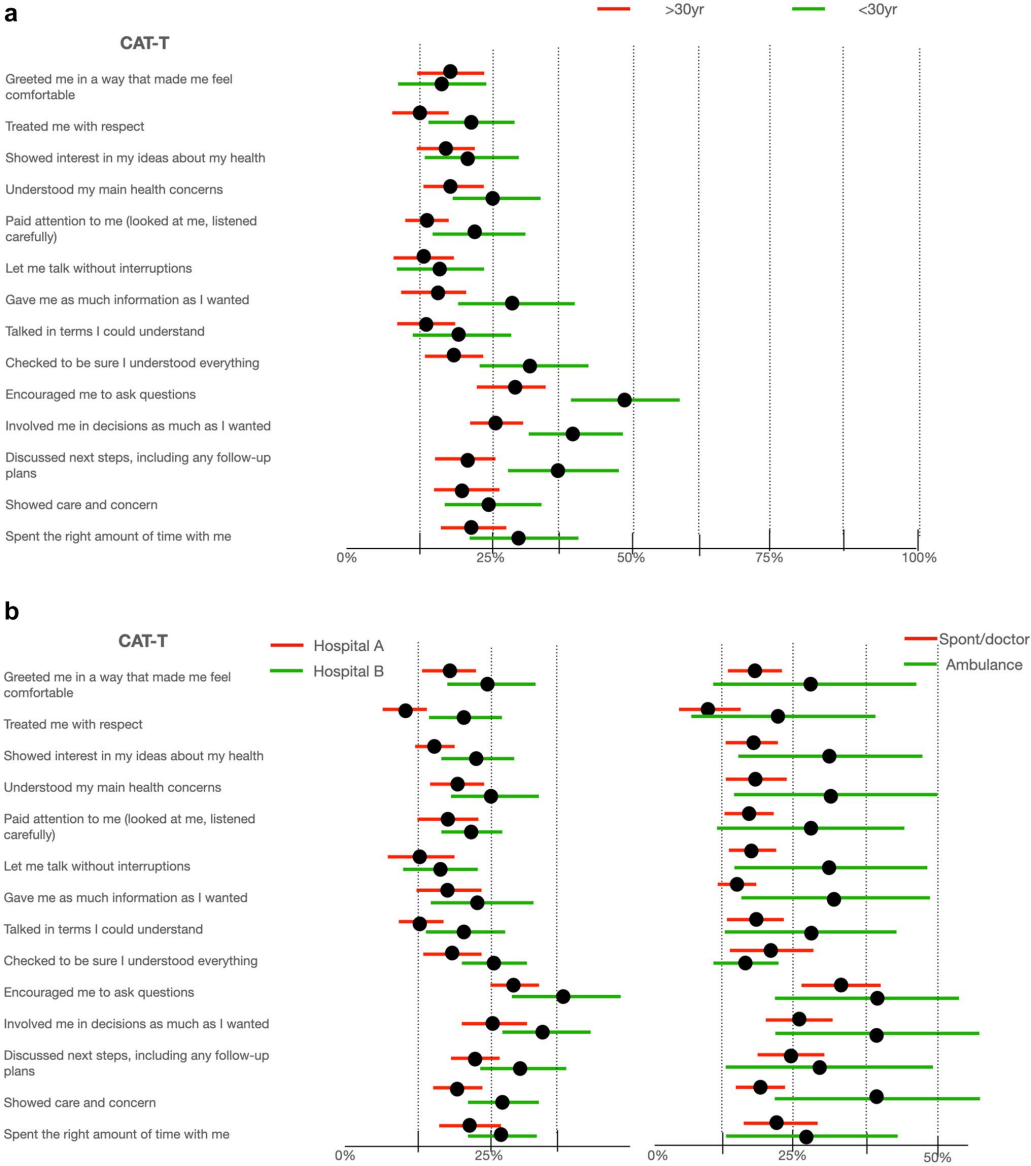
**a** and **b** Comparison of CI of non-satisfied patients between different ages, Hospitals, way of conveyance

Figures 1a and b show the CI of the patients’ answers in relation to the separately collected objective data.

The item with the lowest average score was “the medical team has encouraged me to ask questions”. The two items who reached the highest average score were “the medical team treated me with respect” and “the medical team let me talk without interruptions”.

Comparing the proportion of patients who gave “satisfied” answers with the proportion of patients who gave “non-satisfied” answers the following were statistically significant: young patients under 30 years (less satisfied) vs. adult and elderly patients (more satisfied); patients assessed in hospital A (more satisfied) vs. patients assessed in hospital B (less satisfied); patients conveyed by ambulance (less satisfied) vs. patients who arrived spontaneously or via general practitioner announcement (more satisfied).

Patients assessed by more senior doctors tended to give more “satisfied” answers with respect to patients evaluated by residents, but the difference did not reach statistical difference.

In our study, the long waiting times and weekend days did not generate less satisfied responses.

## Discussion

This study represents one of the few works offering quantitative data on communication efficacy in the ED, among the very few that consider the medical team (there are a little more at the nursing level) and is the work with the largest number of completed questionnaires [15, 16].

The paper links some objective data (age of the patient, hospital where the patient is seen, patients brought to the hospital by ambulance) with the patient’s perception of the communication offered by the medical team.

We observed a lower degree of appreciation in younger patients who tended to be less satisfied with their ED visit as opposed to older patients. This could be because younger individuals tend to be more sensitive towards different aspects of information flow and communication, being more skilled with smart devices and the internet. They may also often have more or higher expectations towards the treatment they will receive in the ED: it is possible that the access to various media (or simply modern culture) influences these expectations and that they already present to the ED with specific diagnostic exams in mind which they expect to be performed [20, 21].

On this topic the literature is debating: De Voe et al. [18] and Mc Carthy et al. [16] found no association between patients’ age and perceptions of communication, while Rotten et al. [19] found a variation in terms of more negative perceptions of communication by younger patients.

With respect to the type of hospital where the patient is seen, the literature is very poor. What appears from the few published studies is that patient satisfaction normally decreases as the size of the hospital increases [22].

Our reality is in fact very peculiar because the same medical and nursing staff work on the two different sites. To explain the fact that the respondents were less satisfied with the communication in the Level II hospital, we hypothesized that patients had different expectations. Indeed, in our city, there is a positive bias towards smaller, relaxing and cosy hospitals in terms of expected services. We recognize that this is just an assumption and should be the subject of a forthcoming study.

The study also outlined significant difference in patients’ perception of medical communication when presenting to ED by ambulance, as opposed to the walk-ins, in particular with regard to the attention given to them by the doctor. In a qualitative study on the transition of care from the prehospital system to the emergency department (a study conducted, however, on nurses), the authors found that a verbal report with involvement of the patient enhanced handovers and provided the opportunity for patients to increase information. On the other hand, a breakdown in communication resulted in the patients’ trust being lost [23, 24]. In our ED, in fact, the patients are usually silenced during the handover between ambulance and ED teams, to let it be swiftly completed. This may create a dynamic in which, though everyone is concerned about the patient’s clinical problem, the patient him/herself may feel neglected in the exact moment when receiving attention could be most reassuring.

In this study, patients appeared more satisfied with communication with senior doctors with more experience. The difference did not reach statistical significance, but there was a trend. Statistical significance may not have been achieved because senior physicians intervene in a smaller number of cases, notably the more complex ones, which are often admitted to the hospital. More data are needed in the future to clarify this aspect.

This trend could be due to the fact that senior physicians tend to be more skilled in targeting a specific diagnostic hypothesis and communicating that to the patient, along with a more linear workup plan. Senior physicians also tend to be more skilled in welcoming the patient, understanding his/her main concern and giving reassurance. On the other hand, it is demonstrated that more experienced doctors are interrupted more often by different tasks and problems of the ED, which is certainly not conductive to communication [25]. It has to be noted that during holidays and weekends, the more senior doctors and specialists are often only available on-call and, in fact, patient satisfaction on these days tends to be lower as compared to week days, even if the statistical significance is not reached (similar data are available in the literature) [26].

There is also an important aspect to be underlined: waiting times did not present differences, both when it came to overall waiting times, and global length of stay in the ED. Other authors [16, 27] have found the same results, and only a qualitative study found the opposite [28]. This, in our view, indicates that communicating well with the patient is of utmost importance.

Lastly, among all the items, the one that consistently received the least good response was "the medical team encouraged me to ask questions". This is also in agreement with what has been found in the literature [16]. A possible explanation can be the limited time the emergency physicians can spend with outpatients due to overcrowding and the concomitant presence of critical patients. The cue for improving this aspect, may be sensitising medical staff to encourage patients to ask questions, particularly with regard to younger patients.

## Limitations

Our study has some limitations: the data is generalised: one-to-one interactions were not qualitatively analysed and we did not consider many other factors (ethnicity, educational level, reason for presenting to the ED…). Among the selection criteria we included only discharged people, preferring to study outpatients. There are many variables that come into play when patients are admitted to the hospital. We also decided not to involve the nursing team, though the nurses have an important role in determining the patients’ perception of the level of communication delivered in the ED.

Finally, our results may be culture dependent and difficult to extrapolate to other places and cultures.

## Conclusions

This study has established that age, setting, way of conveyance of patients to the hospital can play a role in influencing patients’ perception of medical communication in the ED.

These are objective factors which are not strictly tied to communicative skills, and which may influence patients’ perception of communication, thus their satisfaction and experience in the ED.

Overall waiting time and overall length of stay also did not significantly influence patient satisfaction in this study. Although a negative result does not prove the absence of correlation, it does help to emphasize that what matters is that patients ultimately have as positive an experience in the ED as possible. This could consequently improve the overall quality of care.

### Supplementary Information

Below is the link to the electronic supplementary material.

Supplementary file1 (XLSX 833 KB)

## Data Availability

The datasets generated during and/or analyzed during the current study are available from the corresponding author on reasonable request.
